# Secondary Glaucoma Following Corneal Transplantation: A Comprehensive Review of Pathophysiology and Therapeutic Approaches

**DOI:** 10.7759/cureus.69882

**Published:** 2024-09-21

**Authors:** Gayathri Donthula, Sachin Daigavane

**Affiliations:** 1 Ophthalmology, Jawaharlal Nehru Medical College, Datta Meghe Institute of Higher Education and Research, Wardha, IND

**Keywords:** corneal transplantation, corticosteroids, diagnostic techniques, intraocular pressure, secondary glaucoma, therapeutic approaches

## Abstract

Corneal transplantation is a critical surgical procedure aimed at restoring vision in patients with corneal blindness or severe damage. This review focuses on secondary glaucoma, a significant postoperative complication, with the primary objective of providing a comprehensive analysis of its pathophysiology, risk factors, diagnostic challenges, and therapeutic approaches. Unlike other reviews, this work emphasizes the interplay between inflammatory responses, corticosteroid use, and structural changes in the eye that lead to elevated intraocular pressure (IOP) after transplantation. A comprehensive review of the literature was conducted, including studies on postcorneal transplantation glaucoma, to highlight both clinical outcomes and the efficacy of current management strategies. Key findings indicate that medical treatments, such as prostaglandin analogs and beta-blockers, are effective for IOP control in the early stages, while surgical interventions, like trabeculectomy, are often necessary for more advanced cases. Diagnostic challenges, such as the difficulty of accurate IOP measurement posttransplant, are underscored, along with the importance of advanced imaging techniques for the early detection of optic nerve damage. The pathophysiology of secondary glaucoma involves a complex interaction of postsurgical inflammation, steroid-induced complications, and anatomical changes that hinder aqueous humor outflow. Diagnosis requires a combination of tonometry, gonioscopy, and imaging technologies. Management strategies range from pharmacological treatments to surgical options, with a critical focus on balancing IOP control and minimizing risks to graft survival. Clinically, these findings highlight the need for proactive and tailored management of IOP in corneal transplant patients to preserve both graft function and long-term visual outcomes. Future research should focus on improving diagnostic accuracy, developing less invasive surgical techniques, and exploring personalized medicine approaches, including genetic profiling and targeted therapies.

## Introduction and background

Corneal transplantation, or corneal grafting, is a pivotal surgical intervention used to restore vision in individuals suffering from corneal blindness or severe corneal damage [1]. This procedure involves the replacement of a diseased or damaged cornea with a healthy donor cornea. Over the years, advancements in surgical techniques and postoperative care have substantially improved the success rates and visual outcomes of corneal transplantation [1]. The two primary types of corneal transplants are penetrating keratoplasty (PK) and endothelial keratoplasty (EK). PK involves the replacement of the full-thickness cornea, while EK focuses on replacing only the inner endothelial layer, thereby preserving the corneal stroma. Despite these advancements, complications following corneal transplantation, including secondary glaucoma, remain a significant concern [2].

Understanding secondary glaucoma is crucial due to its potential impact on both the success of the corneal transplant and the patient's overall visual health. Secondary glaucoma is characterized by an increase in intraocular pressure (IOP) that occurs as a complication of the transplantation process [3]. Elevated IOP can lead to optic nerve damage and vision loss if not managed effectively. Therefore, a comprehensive understanding of the pathophysiology, risk factors, and management strategies for secondary glaucoma is essential. Addressing this complication proactively can significantly influence patient outcomes and the long-term success of the corneal graft [4].

This review aims to thoroughly examine secondary glaucoma following corneal transplantation. It will explore the mechanisms and risk factors contributing to the development of this condition, review current diagnostic methods and their challenges, and evaluate various therapeutic approaches for managing secondary glaucoma. Additionally, the review will discuss the outcomes and prognosis associated with secondary glaucoma, as well as its impact on corneal graft survival and patient vision. By highlighting these aspects, the review seeks to enhance clinical practice and improve patient care in individuals undergoing corneal transplantation. Furthermore, the review will identify future research directions to advance the understanding and management of secondary glaucoma in this context.

## Review

Methodology

The methodology for this comprehensive review was designed to systematically analyze and synthesize existing literature on secondary glaucoma following corneal transplantation. A structured search of major scientific databases, including PubMed, MEDLINE, Embase, and the Cochrane Library, was conducted to identify relevant studies published until August 2024, with no limitations on the earliest publication date. The search utilized a combination of key terms such as "secondary glaucoma," "corneal transplantation," "intraocular pressure," "penetrating keratoplasty," "endothelial keratoplasty," "steroid-induced glaucoma," "glaucoma diagnosis," and "glaucoma management," along with Medical Subject Headings (MeSH) to capture all pertinent articles. The inclusion criteria for this review focused on peer-reviewed original research articles, clinical studies, and reviews that specifically addressed secondary glaucoma as a complication of corneal transplantation. Only studies published in English were considered, and those that examined the pathophysiology, diagnostic methods, or management strategies for secondary glaucoma were prioritized. Additionally, studies discussing the impact of secondary glaucoma on corneal graft survival and patient visual outcomes were included. Articles excluded from this review comprised non-peer-reviewed literature, editorials, and case reports with fewer than five cases. Studies not directly related to the subject or published in languages other than English were also excluded. Study selection was performed by two independent reviewers, who initially screened titles and abstracts to identify studies that met the eligibility criteria. In cases of disagreement, a third reviewer was consulted to resolve any discrepancies. Full-text articles of potentially relevant studies were reviewed thoroughly to confirm their eligibility. For all studies that met the inlusion criteria, data extraction was performed using a standardized form. This process gathered information on study design, methodology, population characteristics, the type of corneal transplantation performed (e.g., PK or EK), and the primary findings related to secondary glaucoma. The data were then synthesized and analyzed to gain a comprehensive understanding of the topic.

Pathophysiology of secondary glaucoma postcorneal transplantation

Secondary glaucoma is characterized by increased IOP resulting from identifiable underlying causes, distinguishing it from primary glaucoma, which occurs without such factors. This condition is generally classified into two main types: secondary open-angle glaucoma and secondary angle-closure glaucoma [5]. Secondary open-angle glaucoma occurs when the trabecular meshwork is obstructed, impairing the proper drainage of aqueous humor. Conversely, secondary angle-closure glaucoma arises when the angle between the cornea and iris narrows or closes, obstructing the aqueous humor's outflow. Accurate classification is crucial for diagnosing and managing glaucoma effectively, especially in patients who have undergone corneal transplantation [6]. Several mechanisms contribute to the development of secondary glaucoma following corneal transplantation. A significant factor is postsurgical inflammation, which can lead to scarring and obstruction of the trabecular meshwork. This inflammation may result from surgical trauma or an immune response to the transplanted tissue, both of which can elevate IOP [7]. Steroid-induced glaucoma is another critical mechanism. Corticosteroids are commonly prescribed post-surgery to control inflammation, but they can inadvertently raise IOP in susceptible individuals. This occurs due to their effects on aqueous humor dynamics and trabecular meshwork function, leading to secondary glaucoma [8]. Structural changes in the eye also play a crucial role in the pathophysiology of secondary glaucoma. Surgical alterations to the eye’s anatomy, such as changes in the anterior chamber's shape or damage to the trabecular meshwork, can impede aqueous outflow. These structural modifications often result from the specific surgical techniques used during the corneal transplant [4]. Impaired aqueous humor dynamics can significantly contribute to elevated IOP. Disruptions in the normal flow and drainage of aqueous humor may arise from inflammation, surgical trauma, or the presence of foreign tissue. These impaired dynamics are a hallmark of secondary glaucoma [9]. Several risk factors are associated with the development of secondary glaucoma after corneal transplantation. The type of corneal graft performed is a primary factor. The risk of secondary glaucoma can vary depending on whether PK or EK is performed. Generally, PK is associated with a higher incidence of postoperative complications, including glaucoma, compared to EK [10]. Pre-existing ocular conditions also influence the risk of secondary glaucoma. Patients with a history of ocular diseases, such as uveitis, previous glaucoma, or other conditions affecting the eye’s anatomy, are at a higher risk of developing secondary glaucoma after corneal transplantation [6]. Furthermore, the surgical technique and quality of postoperative care are crucial in determining the risk of secondary glaucoma. Techniques that minimize trauma to the trabecular meshwork and effective management of inflammation can reduce the likelihood of elevated IOP post-surgery [11]. Therefore, understanding these mechanisms and risk factors is essential for preventing, detecting, and managing secondary glaucoma in patients undergoing corneal transplantation. Regular monitoring of IOP and diligent postoperative care are vital to mitigating this risk and ensuring optimal patient outcomes [11].

Diagnosis of secondary glaucoma

Diagnosing secondary glaucoma is a complex process involving the evaluation of clinical presentations, the utilization of various diagnostic techniques, and the differentiation from other types of glaucoma [12]. Patients with secondary glaucoma may present with a range of symptoms that vary in severity. Common symptoms include blurred vision, severe eye pain, headaches, nausea, and vomiting. Some individuals may also experience halos around lights. These symptoms often prompt individuals to seek medical attention, which can indicate significant IOP elevation and the potential for optic nerve damage. Recognizing these symptoms is crucial for timely intervention and effective management [6]. Several diagnostic techniques are employed to confirm a diagnosis of secondary glaucoma. Tonometry, which measures IOP, is fundamental in glaucoma assessment. Elevated IOP is a hallmark of glaucoma; precise measurement is essential for diagnosis. Goldmann application tonometry is one of the most commonly used methods for obtaining accurate readings [13]. Gonioscopy is another critical diagnostic tool for examining the anterior chamber angle. This technique enables clinicians to visualize the eye's drainage structures, helping to determine whether the glaucoma is open-angle or angle-closure. Identifying the status of the angle is essential for diagnosing the underlying cause of secondary glaucoma [14]. In addition to tonometry and gonioscopy, advanced imaging modalities such as Optical Coherence Tomography (OCT) and Heidelberg Retinal Tomography (HRT) play a significant role in the diagnostic process. These imaging techniques provide detailed images of the optic nerve and retinal structures, allowing for the assessment of optic nerve damage and the monitoring of changes over time. This information is crucial for effective glaucoma management [15]. Differentiating secondary glaucoma from primary glaucoma is essential for appropriate treatment. Identifiable underlying causes, such as eye trauma, corticosteroid use, uveitis, or neovascularization, characterize secondary glaucoma. In contrast, primary glaucoma occurs without any identifiable cause. This distinction is important because the treatment of secondary glaucoma often involves addressing the underlying condition in addition to managing IOP [4]. Diagnostic modalities for secondary glaucoma following corneal transplantation are summarized in Table 1.

**Table 1:** Diagnostic modalities for secondary glaucoma following corneal transplantation

Diagnostic Modality	Description	Key Features	Clinical Relevance
Tonometry [13]	Measurement of intraocular pressure (IOP)	Elevated IOP (>21 mmHg) is a hallmark of glaucoma	Essential for early detection of increased IOP, which is a key indicator of glaucoma
Gonioscopy [14]	Examination of the anterior chamber angle to assess the drainage angle	Narrow or closed angles, synechiae, or abnormal structures can indicate secondary glaucoma	Helps differentiate between open-angle and angle-closure glaucoma
Optical Coherence Tomography (OCT) [16]	Non-invasive imaging that provides high-resolution images of the optic nerve and retina	It can show thinning of the retinal nerve fiber layer (RNFL) and other structural changes in the eye	Useful for monitoring progressive optic nerve damage
Heidelberg Retina Tomograph (HRT) [17]	Imaging modality that provides a 3D topographic image of the optic nerve head	Assesses optic nerve head contour and detects changes in the optic nerve over time	Valuable for detecting structural changes in the optic nerve head
Visual Field Testing [18]	Functional tests to assess the patient's field of vision and detect any loss of peripheral vision	Visual field defects (e.g., arcuate scotomas) are common in glaucoma	Important for evaluating functional impairment in glaucoma patients
Pachymetry [19]	Measurement of corneal thickness	Thin corneas can lead to underestimation of IOP, while thick corneas may cause overestimation	Critical for accurate IOP measurement and glaucoma risk assessment
Slit-Lamp Examination [20]	A detailed examination of the anterior segment of the eye	Detects signs of inflammation, corneal edema, synechiae, or changes in the iris	Helps identify contributing factors such as postsurgical inflammation
Ultrasound Biomicroscopy (UBM) [21]	High-frequency ultrasound imaging of the anterior segment	Provides detailed images of the anterior segment, including the ciliary body and anterior chamber	Useful in cases where gonioscopy is not conclusive or when structural abnormalities exist

Management and therapeutic approaches

The management of secondary glaucoma following corneal transplantation involves a comprehensive approach that integrates medical and surgical strategies, individualized treatment plans, and meticulous postoperative care [1]. Medical management is typically the initial approach for treating secondary glaucoma. This includes both topical and systemic pharmacological therapies. Topical treatments commonly used to lower IOP include prostaglandin analogs, beta-blockers, alpha agonists, carbonic anhydrase inhibitors, and Rho kinase inhibitors. In cases where topical treatments are inadequate, systemic treatments such as oral carbonic anhydrase inhibitors may be employed. Managing elevated IOP while balancing the risk of steroid-induced glaucoma requires careful consideration of anti-inflammatory and anti-glaucoma medications [22]. When medical therapy fails to control IOP sufficiently, surgical management is considered. Glaucoma filtration surgery, such as trabeculectomy, creates a new drainage pathway for aqueous humor to bypass the trabecular meshwork, lowering IOP. The success of trabeculectomy may depend on the absence of inflammation and the overall health of the eye post-transplant. When trabeculectomy is not feasible or has proven unsuccessful, implanting drainage devices (e.g., Ahmed or Baerveldt implants) offers an alternative route for aqueous drainage, particularly in eyes with previous surgeries or significant scarring [23]. Treatment plans should be individualized, considering factors such as the severity of glaucoma, other ocular conditions, and the patient's overall health. Combination therapy, which involves using multiple medications, can improve IOP control, especially in cases where single agents are insufficient. For instance, combining a prostaglandin analog with a beta-blocker can enhance therapeutic effects [24]. Following medical and surgical interventions, ongoing monitoring of IOP and visual function is crucial. This enables timely adjustments to the treatment plan and assessment of potential complications, such as infection, bleb failure, or further elevation of IOP. Educating patients about the importance of adhering to prescribed medications and attending follow-up appointments is vital for successful long-term management [25]. Management and therapeutic approaches for secondary glaucoma following corneal transplantation are summarized in Table 2.

**Table 2:** Management and therapeutic approaches for secondary glaucoma following corneal transplantation

Therapeutic Approach	Description	Indications	Advantages	Limitations
Pharmacological Management [26]	Use of anti-glaucoma medications to reduce intraocular pressure (IOP). Includes beta-blockers, prostaglandin analogs, carbonic anhydrase inhibitors, and alpha agonists	Elevated IOP due to secondary glaucoma. First-line treatment option for mild to moderate cases	Non-invasive, easily adjustable dosage, effective for controlling IOP in many patients	Potential side effects include ocular irritation, systemic side effects, and reduced efficacy over time
Steroid Sparing Agents [27]	Alternative anti-inflammatory agents to reduce steroid-induced glaucoma	Patients with steroid-induced secondary glaucoma following corneal transplantation	Helps reduce dependency on steroids while maintaining anti-inflammatory effects	Limited efficacy in controlling inflammation for all patients
Glaucoma Filtration Surgery [28]	A surgical procedure is needed to create an alternative drainage pathway to lower IOP (e.g., trabeculectomy)	Patients with uncontrolled IOP or progressive optic nerve damage despite medical management	It can provide long-term control of IOP and reduce the need for medications	Risk of infection, hypotony, bleb failure, and complications may affect corneal graft survival
Implantation of Drainage Devices [29]	Use glaucoma drainage devices (e.g., Ahmed valve, Baerveldt implant) to divert aqueous humor	Patients with advanced secondary glaucoma or failed filtration surgery	Effective in maintaining long-term IOP control in patients resistant to other treatments	Device-related complications such as tube erosion, exposure, and impact on corneal graft clarity
Cyclodestructive Procedures [30]	Procedures like cyclophotocoagulation reduce aqueous humor production by targeting the ciliary body	Reserved for refractory cases or eyes with poor visual prognosis	Reduces IOP without the need for invasive surgery	This can result in significant complications such as hypotony and inflammation, potentially affecting grafts
Combined Therapies [31]	Use of both medical and surgical treatments to control IOP and inflammation	Patients with complex secondary glaucoma require both IOP control and inflammation management	It can address multiple causes of secondary glaucoma with a tailored, multifaceted approach	There is an increased risk of side effects from combining therapies and the need for close response monitoring
Postoperative Monitoring and Care [32]	Regular follow-up visits, IOP monitoring, and visual field tests to detect and manage glaucoma progression	All patients postcorneal transplantation to identify early signs of secondary glaucoma	Early detection of complications improves treatment outcomes	Requires long-term commitment from both patient and clinician, as secondary glaucoma can develop over time

Outcomes and prognosis

Outcomes and prognosis for secondary glaucoma following corneal transplantation are crucial for understanding their impact on patient health and visual function. Secondary glaucoma can significantly affect corneal graft survival, as elevated IOP may lead to optic nerve damage and potentially compromise the health of the corneal graft [3]. Research indicates that uncontrolled IOP is linked to a higher risk of graft rejection and failure, as increased pressure can disrupt the delicate balance necessary for graft integration and healing. Therefore, prompt management of IOP is essential to preserve both the graft and the patient's vision [10]. The long-term visual and functional outcomes for patients with secondary glaucoma post-coronal transplantation can vary widely. Some patients may maintain good visual acuity with effective IOP management, while others might experience significant vision loss due to progressive optic nerve damage. Prognosis often depends on factors such as the underlying cause of secondary glaucoma, the timing of the intervention, and the effectiveness of the treatment strategies employed. Regular monitoring and timely treatment are critical for optimizing visual outcomes, as early detection of elevated IOP can prevent irreversible damage [33]. To improve prognosis in patients with secondary glaucoma following corneal transplantation, several strategies should be considered. Early detection and management are paramount; regular eye examinations after surgery can help promptly identify elevated IOP, enabling timely intervention [34]. Additionally, targeted treatment tailored to the underlying cause of secondary glaucoma is vital. This may involve medications to lower IOP, laser therapies, or surgical interventions if necessary. A multidisciplinary approach involving collaboration between ophthalmologists and other healthcare providers can enhance overall patient management, especially in cases where systemic conditions contribute to secondary glaucoma [34]. Patient education is also a key factor in improving outcomes. Educating patients about the importance of adhering to treatment regimens and attending regular follow-up appointments can significantly impact their prognosis. While secondary glaucoma poses a serious risk to corneal graft survival and visual function, timely intervention and comprehensive management strategies can markedly improve prognosis. Regular monitoring and individualized treatment plans are essential for optimizing patient outcomes [35].

Future directions and research

Future directions in managing secondary glaucoma are evolving, focusing on advances in diagnostic technologies, novel therapeutic approaches, and the potential for personalized medicine. These developments promise to enhance the detection, treatment, and overall management of this complex condition [36]. Recent advancements in diagnostic technologies have greatly improved the detection and monitoring of secondary glaucoma. Notably, OCT provides high-resolution cross-sectional images of the retina and optic nerve, allowing for a detailed assessment of structural changes associated with glaucoma. This technology facilitates early detection and ongoing monitoring of disease progression [37]. Additionally, improvements in automated perimetry have increased its sensitivity, enabling the detection of subtle vision changes that may indicate worsening glaucoma. Gonioscopy remains the gold standard for evaluating the anterior chamber angle, which is crucial for distinguishing between primary and secondary angle-closure glaucoma. Newer gonioscopic techniques and imaging modalities are being developed to quantify angle characteristics more effectively, further aiding diagnosis [38]. Therapeutic strategies for secondary glaucoma are becoming increasingly sophisticated, with several novel approaches on the horizon. Minimally invasive glaucoma surgery (MIGS) offers safer alternatives to traditional surgeries, with reduced recovery times and fewer complications [39]. Techniques such as trabecular micro-bypass stents are particularly promising for managing secondary open-angle glaucoma. Additionally, targeted drug delivery systems, including sustained-release implants, are being explored to provide consistent IOP control while minimizing systemic side effects associated with traditional therapies. Research into combination therapies that utilize medications targeting different pathways involved in IOP regulation aims to enhance efficacy and improve patient adherence to treatment regimens [39]. The concept of personalized medicine is gaining traction in glaucoma management, which could significantly improve outcomes for patients with secondary glaucoma. Genetic profiling may help identify individuals predisposed to developing secondary glaucoma, allowing for tailored treatment plans that consider specific risk factors [40]. By understanding a patient's unique clinical profile and response to previous treatments, ophthalmologists can create more effective and individualized management strategies. Ongoing research into biomarkers that could predict disease progression or response to treatment holds the potential for proactive adjustments to therapy based on individual patient needs [40]. Future directions and research in managing secondary glaucoma following corneal transplantation are summarized in Table 3.

**Table 3:** Future directions and research in the management of secondary glaucoma following corneal transplantation

Area of Research	Description	Potential Impact	Current Challenges
Advances in Diagnostic Technologies [41]	Development of novel imaging modalities and artificial intelligence (AI)-based algorithms for early detection of secondary glaucoma	Improved accuracy in diagnosing secondary glaucoma at earlier stages, better prediction of disease progression	High cost of advanced diagnostic tools and integration into clinical practice
Personalized Medicine Approaches [42]	Tailoring treatment plans based on genetic, molecular, and individual patient factors	More effective, individualized treatments that minimize side effects and improve outcomes	Lack of large-scale data and clinical trials to support personalized medicine in glaucoma treatment
Biomarker Discovery [43]	Identification of specific biomarkers for early detection and risk stratification of secondary glaucoma	Earlier diagnosis and targeted therapy potentially reduce the need for invasive procedures	There is a need for further research to validate potential biomarkers and their clinical applicability
Stem Cell Therapy and Regenerative Medicine [44]	Investigating stem cell treatments to repair damaged ocular tissues and restore function	Potential to regenerate damaged trabecular meshwork or optic nerve, offering new treatment avenues	Ethical concerns, regulatory hurdles, and lack of long-term data on safety and efficacy
Gene Therapy [45]	Exploring gene therapy to prevent or reverse glaucoma by targeting specific genetic factors	It could offer a curative approach to glaucoma by directly addressing underlying genetic causes	Delivery mechanisms, long-term efficacy, and safety concerns remain significant barriers
Novel Drug Development [46]	Research into new classes of drugs, such as neuroprotective agents and drugs targeting aqueous humor dynamics	It could provide more effective and safer options for intraocular pressure (IOP) control and neuroprotection	High costs of drug development, clinical trial design, and regulatory approval processes
Minimally Invasive Surgical Techniques [28]	Development of less invasive surgical methods for managing secondary glaucoma	Reduced risk of complications, shorter recovery time, and better patient outcomes	Requires further refinement and long-term follow-up data to assess the efficacy of these techniques
Long-Term Outcomes of Combination Therapies [47]	Studying the effectiveness of combining medical, surgical, and alternative treatments over extended periods	Improved long-term control of IOP and preservation of corneal grafts	Need for larger, multicenter trials to evaluate combination approaches in diverse populations
Artificial Intelligence (AI) in Glaucoma Management [48]	AI-driven tools to assist in diagnosis, treatment planning, and monitoring of disease progression	Enhanced decision-making for clinicians, leading to earlier intervention and better patient outcomes	Integration of AI into clinical settings, data privacy, and validation of AI algorithms in real-world practice

## Conclusions

This review highlights the significant impact of secondary glaucoma on both visual outcomes and corneal graft survival following corneal transplantation. Key findings reveal that elevated IOP, driven by postsurgical inflammation, steroid use, and anatomical changes, poses a critical risk for optic nerve damage and graft failure if not properly managed. The review underscores the importance of a multifaceted approach, combining accurate diagnostic methods, such as tonometry and advanced imaging, with targeted therapeutic strategies ranging from medical treatments to surgical interventions. Clinically, these findings emphasize the need for proactive and individualized IOP management in corneal transplant patients. Early diagnosis through regular monitoring and the use of advanced diagnostic tools is crucial to preventing optic nerve damage and preserving graft function. Clinicians should consider revising management strategies, particularly by balancing IOP control with minimizing the adverse effects of steroids. Surgical options should be considered for patients unresponsive to medical treatments, and tailored approaches based on the specific pathophysiology of each case are recommended.

Future research should focus on improving diagnostic accuracy, particularly through advancements in imaging technologies and minimally invasive procedures. Studies exploring personalized medicine approaches, such as genetic profiling to identify high-risk patients, are also needed. Additionally, research into the long-term outcomes of combination therapies and the development of novel treatments targeting the underlying mechanisms of secondary glaucoma could further enhance patient outcomes. Preventing and managing secondary glaucoma should be a primary focus for clinicians working with corneal transplant patients. Emphasizing early detection and individualized care can significantly improve both visual and functional outcomes. Ultimately, this review confirms its original objective of providing a comprehensive understanding of secondary glaucoma, offering actionable insights that can be integrated into clinical practice to optimize care for corneal transplant recipients.
